# Consumer genomics will change your life, whether you get tested or not

**DOI:** 10.1186/s13059-018-1506-1

**Published:** 2018-08-20

**Authors:** Razib Khan, David Mittelman

**Affiliations:** 1 Insitome Inc., Austin, TX 78702 USA; 2 Department of Biology, Texas A&M University, College Station, TX 77843 USA

## Abstract

With more than 10 million genotyped customers, the consumer genomics industry is maturing and becoming a mainstream phenomenon. At last, innovations and applications, some unforeseen, are being brought to the masses.

## Introduction

**Fig. 1:**
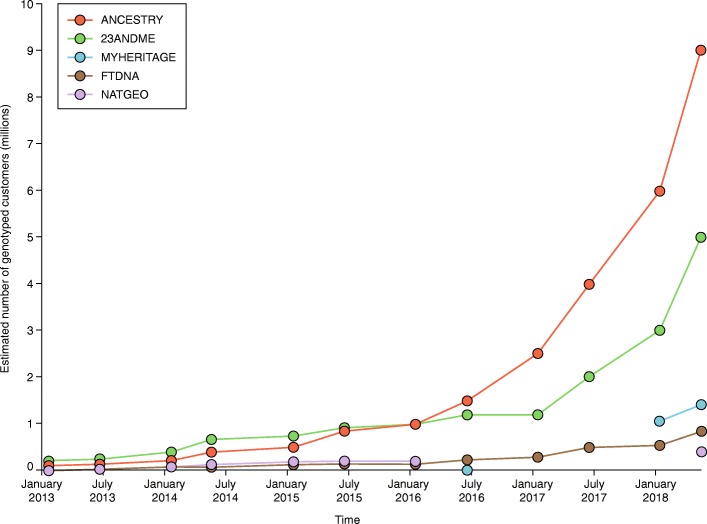
Consumer genomics is in the exponential growth phase. The estimated number (in millions) of genotyped customers from various consumer genomics companies over the past 5 years. These estimates were pulled from the ISOGG Autosomal DNA Testing Comparison Wiki (https://isogg.org/wiki/Autosomal_DNA_testing_comparison_chart), following an approach similar to that first used by Leah Larkin. These estimates are also consistent with another recent report by Yaniv Erlich et al. [2]

At the start of this year, the direct-to-consumer personal genomics industry surpassed 10 million genotyped consumers [1, 2], a ten-fold increase since our last comment in *Genome Biology* on the state of consumer genomics [3]. Between the end of 2013 and 2016 there was steady growth in consumer numbers, but after 2016 the sector began to grow exponentially (Fig. 1). Looking forward, we could project another ten-fold increase by 2021, with upwards of 100 million genotyped individuals. It is likely that the growth rate will exceed these projections because of increased advertising spend from market leaders and decreasing genotyping and sequencing costs. In the next few years, more companies will enter the direct-to-consumer (retail) market for genomics. We are a long way from saturation on the S-shaped growth curve, especially with the realization of new market opportunities, which span prediction of medical risks, precise genealogical reconstructions, and even crime solving.

These enormous numbers of genotyped consumers will generate massive returns on scale, allowing for greater innovation and insight. If hundreds of millions of consumers contribute to genetic databases, then the power of genealogical algorithms to infer matches will increase, until the likelihood of matching a relative, *if* you have close relatives (at least in the United States), will converge upon total certainty [4]. Public databases such as GEDMatch now include data from one million samples, sufficient to predict a 90% chance of finding at least one third-cousin relative. Even with this ‘small’ database, consumers will almost certainly find relatives, and many of them. Genealogy has proved itself to be a sector with an affluent and passionate consumer base, as evidenced by the multibillion dollar valuation of the Ancestry online database thanks to millions of discretionary subscriptions [5].

The huge numbers of genotypes provided by consumers are valuable for genealogy, but as the numbers of genotypes increase into the millions, the data become even more valuable for trait prediction and medical applications. The large sample sizes allow for greater statistical power to detect genome-wide associations, which may be useful in linking genomic markers to functional traits and clinical phenotypes. 23andMe, for example, has amassed a database with sample numbers in the millions with which they are now working to obtain genotype–phenotype associations [6]. The analysis of rare variations becomes immensely powerful when sample sizes approach a hundred million genotypes, and medicine could be truly personalized when such massive information reservoirs are available. We simply do not know what we might be able to do until we hit those sample sizes, as that is still unexplored territory.

## New technology platforms will help us to get more from consumer genomics

All this brings us to emerging improvements in analysis technologies and platforms for consumer genomics. One of the new entrants is Helix, a consumer-focused genomics startup backed by Illumina. Utilizing exome sequencing, supplemented with single nucleotide polymorphism (SNP) positions that span the genome, Helix aims to go beyond the SNP-chip paradigm championed by current market leaders such as Ancestry and 23andMe. Meanwhile, Veritas Genetics, a well-funded Cambridge, MA, startup aims to take whole-genome sequencing (WGS) to the public. Fifteen years after the Human Genome Project, which cost about $3 billion dollars, Veritas is commercializing a retail product that provides a high-quality sequence for $1000. Gencove, a startup based in New York City, takes a middle ground between SNP arrays and full genome sequencing. Gencove has developed an affordable low-coverage WGS assay that represents an improvement over current SNP array technology, with coverage under 1×. The low-coverage sequencing assay is paired with imputation to deliver a product that is not only competitive but has advantages over SNP arrays in terms of cost and avoidance of ascertainment bias. We suspect that the SNP array era of the 2010s will be superseded by the sequencing decade of the 2020s.

Although the number of genotyped and sequenced consumers has increased greatly, the services and products offered to make sense of their data remain curiously limited. This deficit might be a function of the late 2013 Food and Drug Administration (FDA) debacle with 23andMe, which cast a pall over the whole sector. Prior to the end of 2013, 23andMe had been providing a wide array of medically relevant analyses. In 2013, the FDA told the company that they could no longer do this, as these were unapproved ‘medical tests’. The firm could only provide information on ancestry and nonmedical traits to their future customers. Fortunately, over the past few years, 23andMe has established an avenue of communication with the FDA and, more importantly, a path for FDA approval for its tests [7]. 23andMe’s carrier screening markers are now FDA approved. The future looks bright, if still not totally certain.

Smaller companies such as Promethease, which leverage raw data generated by 23andMe, Ancestry, and others, continue to occupy a niche in analyzing genetic information. Using public data and stitching it together in a rough and basic user interface, Promethease and other such companies aim to eliminate the intermediaries between the consumer and the data. Instead of an institution or geneticist serving as a guide, these companies aim to educate the public in a raw and direct fashion, albeit with some controversy.

Helix and Gencove take a third approach. Instead of providing the full stack of services or leveraging the raw data provided by third parties, these two companies want to be technology platforms for other independent players and foster an ecosystem of analysis applications for consumers. Helix seems to be modeling its ecosystems on the iTunes store, where Apple hosts and approves applications for consumers who already trust their brand and utilize their hardware platform. The difference here is that Helix is not an established brand and does not yet have a dedicated customer base. Remember that when the iTunes Store debuted in 2008, Apple was not a new company; it had sold more than 5 million computers and more than 50 million iPods.

The need for a strong brand is probably one reason why Helix partnered with National Geographic, whose legacy reaches back over a century. In addition, it is partnering with blue-chip institutions such as the Mayo Clinic and with innovative companies such as Invitae. However, establishing a brand and a customer base are only part of the challenge of growing a successful app store. There are near infinite things you can do with a mobile computer, a camera, and a variety of sensors. There are far fewer apps that can be built on top of a genetic sequence, at least at present. Although the genomics space is in some ways less flexible than the applied engineering that drove innovation in Silicon Valley, it is also uncharted territory. There are still many variants to be discovered, structural features in the genome to be mapped, and parts of a genuinely complete human genome to be sequenced [8]. As these uncertainties are resolved, new applications will undoubtedly appear. After all, applied science depends on discovery in basic science. Perhaps the most exciting aspect is the possibility of discovering whole new applications that were unanticipated.

## Growth of consumer genomics has yielded new and emerging applications

To date, the existing consumer genomics companies have apps that naturally segment into three classes: ancestry, entertainment, and medical. Medical applications often have the upside of being actionable, but they are also subject to regulatory oversight. Furthermore, there are limited medical insights to be gained today through genotyping or sequencing alone. Entertainment, ancestry, and genealogy are more loosely regulated, but they suffer from a perception that they are not an essential aspect of a person’s lifestyle. Entertainment apps often seem trivial or frivolous. But entertainment is important to allow us to flourish and for establishing connections. It is a something people are willing to spend money on because it is deeply rewarding. The key is not to undermine the credibility of the hard science while creating something fun and engaging. Not all apps have to provide a genome interpretation. Apps that leverage genetics can result in an efflorescence of cultural and entertainment possibilities previously not imaginable. For example, clothing and apparel that signals one’s genetic status or lineage is becoming more common. The ‘Haplotee’ offered by DNAGeeks.com is an example of genetics-inspired apparel. The shirt features your Y-DNA or MT-DNA haplogroup, along with a map of the related ‘Out of Africa’ migration pattern for the haplogroup. Furthermore, the Helix App Store has additional apparel items, including personalized scarves that allow customers to showcase their individuality through clothing and accessories [9].

Most recently, the arrest of the alleged Golden State serial killer in California using the methods of genetic genealogy shows how surprising and novel outcomes can be the product of disparate technological movements occurring in parallel [10]. Now that the law enforcement community is aware of the possibilities, we are likely to see a major rise in the success resolution of criminal cases in the near future.

## Final thoughts

We see the future through a dark mirror, but the current prediction for WGS is that 60 million Americans will be sequenced by 2025. Alongside the SNP array data, this sequencing will contribute to an enormous database that one can utilize to provide value to consumers. Until then, the next four years will see the SNP array and exome-based companies expand their markets at least geometrically, and perhaps even exponentially if there is more technological innovation. These certainties determine the parameters of the American and international market. But what sells and what does not; well, that is to be determined. The products and services that consumer genomics firms seeded in the SNP array era are likely to be the foundations of the WGS revolution that will come out into the open in the 2020s.

## Abbreviations

FDA: Food and Drug Administration
SNP: Single nucleotide polymorphism
WGS: Whole-genome sequencing
